# Can a Clinic-Based Community Health Worker Intervention Buffer the Negative Impact of the COVID-19 Pandemic on Health and Well-Being of Low-Income Families during Early Childhood

**DOI:** 10.3390/ijerph20146407

**Published:** 2023-07-20

**Authors:** Taylor Salaguinto, Yasmin Guzman, Sarah J. Lowry, Kendra Liljenquist, Rachel LaFontaine, Janette E. Ortiz, Peter G. Szilagyi, Kevin Fiscella, Marcia R. Weaver, Tumaini R. Coker

**Affiliations:** 1 Seattle Children’s Research Institute, Seattle Children’s, Seattle, WA 98101, USA; 2 Department of Pediatrics, School of Medicine, University of Washington, Seattle, WA 98109, USA; 3 Department of Pediatrics, University of California, Los Angeles, CA 90095, USA; 4 Department of Family Medicine, University of Rochester, Rochester, NY 14611, USA; 5 Departments of Health Metrics Sciences & Global Health, University of Washington, Seattle, WA 98121, USA

**Keywords:** COVID-19 impact, pandemic, postpartum, well-being

## Abstract

We examined changes in self-reported mental health, physical health, and emotional support among low-income parents with children ages 0–2 years old from pre-pandemic to pandemic periods and compared changes in parental health among parents who did versus did not have access to a clinic-based community health worker intervention supporting parents at early childhood preventive care visits. We utilized longitudinal parent survey data from pre-COVID-19 and COVID-19 time periods from both the intervention and control arms of an existing cohort of parents enrolled in a 10-clinic cluster randomized controlled trial (RCT). At enrollment (pre-pandemic) and 12-month follow-up (pandemic), participants reported on mental health, physical health, and emotional support using PROMIS measures (n = 401). During the pre-pandemic portion, control and intervention group parents had similar mean T-scores for mental health, physical health, and emotional support. At follow-up, mean T-scores for mental health, physical health, and emotional support decreased across both control and intervention groups, but intervention group parents had smaller declines in mental health T-scores (*p* = 0.005). Our findings indicate that low-income parents with young children suffered significant declines in mental and physical health and emotional support during the pandemic and that the decline in mental health may have been buffered by the community health worker intervention.

## 1. Introduction

COVID-19 has had a disproportionate impact on low-income racial and ethnic minoritized populations [1]. During the COVID-19 pandemic, individuals in communities of color and those living in under-resourced settings have suffered disproportionately from severe disease and high morbidity and mortality from COVID-19 [2,3,4]. However, the full impact that the pandemic has had on the ongoing health and well-being of children and their families, particularly those living in poverty and of minoritized race and ethnicity, extends well beyond COVID-19 specific morbidity and mortality, to general health and well-being [5]. The potential long-term impact of the COVID-19 pandemic on children and families can be described using the socioecological model of development across the course of life, a conceptual model that serves as a guide to understanding how various systems and elements impact health across the course of life [5,6]; this conceptual model has been previously used in a National Academies of Science, Engineering, and Medicine’s consensus report examining the long-term impact of the pandemic on children and families.

A national cross-sectional study of parents demonstrated substantial negative impact of the pandemic on parent and child well-being [7]. A more focused understanding of the pandemic’s impact on parental health and well-being in households with young children in racially and ethnically minoritized communities is needed [8], given the pre-existing systemic inequities in family income, housing, healthcare, employment, and education that these communities have faced due to structural racism in the U.S. [9] and the increased vulnerability that occurs during the early childhood period for families [10,11]. For example, racial and ethnic minority mothers are at greater risk of postpartum depression and report lower levels of social support and greater declines in income during the first postpartum year, compared to white mothers [10,11,12].

There is a critical need to understand how the pandemic affected families with young children; however, data are limited, as most studies have focused on families with school-aged children or employed cross-sectional designs [7,13,14]. Our objectives were to (1) examine changes in the mental, physical, and emotional health of parents with children ages 0–2 years old and (2) examine whether these changes varied for parents who had access to a clinic-based community health worker intervention that supports parents at early childhood preventive care visits. To accomplish this, we used longitudinal parent survey data from pre-COVID-19 and COVID-19 time periods in an existing cohort of low-income, racially and ethnically diverse parents with infants ≤12 months of age at federally qualified health centers (FQHCs) in two states.

## 2. Methods

### 2.1. Data Collection

This study was conducted with participants from the PARENT (Parent-engaged Redesign of Encounters, Newborns to Toddlers) trial (NICHD R01 HD088586) [15], which is a two-state, cluster RCT (clustering on primary care practice) of a team-based approach to preventive pediatric care. Adult parents or legal guardians who spoke English or Spanish and had a child age ≤12 months arriving for a well-child care visit at one of the 10 participating federally qualified health centers (FQHCs) clinics in Los Angeles county, California, and Pierce county, Washington, were eligible for enrollment in the trial. Pre-COVID-19 data collection was in-person, and participants enrolled into the trial were approached prior to their child’s well-child visit. A total of 914 parent participants were enrolled in the PARENT trial. This analysis focuses on the 401 participants from both study arms who were enrolled pre-pandemic, prior to March 2020 (enrolled March 2019–February 2020) and completed a 12-month follow-up survey during the pandemic period. Twelve-month follow-up data were collected over the phone or online, in English or Spanish (March 2020–February 2021). The study was approved by the Seattle Children’s Institutional Review Board on 1 July 2020 (STUDY00002666).

### 2.2. Intervention

Parents who attended a clinic randomized to intervention had their well-child care provided through the PARENT intervention, which uses a PARENT coach (community health worker role) as part of the well-child visit team. The coaches were bilingual, bicultural Latina community health workers who provided anticipatory guidance, psychosocial and social needs screening and referral, and developmental/behavioral surveillance, screening, and guidance at well-visits, in partnership with the primary care provider.

### 2.3. Main Outcomes

#### 2.3.1. Parent Global Physical Health, Mental Health, and Emotional Support

We utilized the Patient-Reported Outcomes Measurement Information System (PROMIS), a set of person-centered validated measures that are freely available, developed with rigorous methods, and used internationally. For parental health, we used the PROMIS Adult Global Physical and Mental Health measures, each having two items and providing an overall assessment of physical and mental health, administered in English and Spanish. The PROMIS Global Physical Health measure assesses adult physical health and the participant’s ability to carry out every day physical activities. The PROMIS Global Mental Health measure asks about general mental health and satisfaction with social activities and relationships. We also used the PROMIS Emotional Support 4-item scale that asks participants the extent to which they have someone to listen to them when they need to talk, someone to confide in, someone who makes them feel appreciated, and someone to talk with when they have had a bad day. PROMIS measures were converted from summed raw scores to T-score values as per the PROMIS scoring manual [16]. Specifically, PROMIS mental health and physical health measures were calculated by taking the sum of 2 questions each on a 5-point scale, for a possible total of 2–10, then converting those sums to corresponding PROMIS T-scores, with values ranging from 25.8 to 64.6 (MH) and from 23.4 to 63.3 (PH). PROMIS emotional support was calculated by taking the sum of 4 questions, each on a 5-point scale, for a total possible of 4–20, then converting those sums to corresponding PROMIS T-scores, for T-score values ranging from 25.7 to 62.0 [16].

#### 2.3.2. Open-Ended Question

Parents were sent an additional survey between August 2020 and June 2021 that focused on their experiences during the pandemic, in which they were asked one open-ended question “Is there anything else that you would like to share about how the COVID-19 pandemic has impacted you and your family?” to help us contextualize the quantitative responses. Participants were free to share as much or as little for this question as they wanted. Because this was a one-item question, research assistants transcribed the parent responses directly into REDCap during the survey administration, capturing word-for-word the parents’ brief responses whenever possible. For parents who completed the survey online, they typed their own responses into the online survey.

#### 2.3.3. Sample Size

For the main trial, we estimated that we would have 80% power to detect meaningful differences on our main study primary outcomes (anticipatory guidance score and proportion of participants with ≥2 ED visits) [15]. This current analysis was a post hoc analysis not included in a priori power calculations as the COVID pandemic started well after the study was conceptualized, planned, and started.

### 2.4. Analysis

#### 2.4.1. Quantitative Responses

We summarized baseline characteristics of the study population overall and by study arm, as well as PROMIS measure outcomes by study arm at 12 months. We calculated each individual’s change in each PROMIS score from baseline (pre-pandemic) to 12 months (during pandemic) in order to carry out a difference-in-differences analysis of changes in PROMIS outcomes over time by study arm. We estimated differences between study arms in change-since-baseline in PROMIS T scores using mixed effects linear regression with study arm as the independent variable and a random effect for clinic. Regression analyses were adjusted for child age due to observed differences between study arms at baseline, both in the full sample (n = 501) and in the subset who responded to the 12-month survey (n = 401). To handle missing data, we compared baseline characteristics of participants missing 12-month primary outcome data to those not missing data and compared those missing primary outcome data in intervention group vs. control group [15]. We used complete case analysis to handle missing data in regression analyses. This approach assumes data missingness at random; results may be biased if missingness is not at random [17].

#### 2.4.2. Qualitative Responses

Three authors conducted the analyses using thematic qualitative analysis. Two members of the research team independently read through all responses (TS and YG) and created codes to represent the major themes that arose from the responses. One author read all responses in English and Spanish, and the second author read responses in English (using translated versions of the Spanish responses). Using an iterative process, we developed a codebook from these codes and then independently coded all responses using the codebook. Discrepancies were discussed with modification of the codebook with the two coders and the principal investigator. We performed thematic analysis of the 111 unique responses that covered a variety of COVID-19 pandemic experiences. We identified salient themes, as well as specific topics that emerged from multiple participants.

## 3. Results

Adult parents or legal guardians (n = 501) with a child age ≤12 months were enrolled in the main PARENT trial prior to 1 March 2020; 93% were mothers; 67% were Latinx; 44% were born outside of U.S.; 45% reported the primary language spoken in their home was a language other than English; and 96% had Medicaid insurance for their child (Table 1). Of those, 401 completed the 12-month follow-up pandemic period survey (response rate 80%). Compared to the larger sample of trial participants, the 401 survey respondents were more likely to be non-US born and report Spanish as their primary language.

### 3.1. Pre-COVID-19 to COVID-19 Changes in Parent Mental Health, Physical Health, and Emotional Support

At baseline (pre-pandemic), control and intervention group parents had similar mean T-scores for Global Mental Health (54.6 vs. 54.8), Global Physical Health (51.4 vs. 51.7), and Emotional Support (57.7 vs. 58.2). At 12-month follow-up (pandemic period), mean T-scores for Global Mental Health (50.8 vs. 53.0), Global Physical Health (49.7 vs. 51.1), and Emotional Support (55.5 vs. 56.2) decreased in both control and intervention groups (Table 2).

### 3.2. Intervention Effect on Pre-COVID-19 to COVID-19 Changes in Parent Mental Health, Physical Health, and Emotional Support

At 12-month follow-up, the intervention group mean T-score for Global Mental Health was significantly higher (53.0, SD 8.0), compared with the control group mean T-score (50.8, SD 7.8), and it had decreased less since the pre-pandemic baseline measurement (decrease of 1.6 points (SD 7.7) vs. 3.3 points (SD 8.7)). Differences in the mean scores for Global Physical Health and Emotional Support were not significantly different between intervention and control at 12-month follow-up. In adjusted analyses, the intervention was associated with an approximately two-point smaller decrease in Global Mental Health mean T-score compared to the control group (Adjusted Difference in Difference 1.87 (CI 0.26–3.49), Table 2).

### 3.3. Open-Ended Question

Some 179 of 501 parents completed the COVID-19-impact survey (response rate 36%). There were 111 unique responses to the open-ended question. Responses varied in length. The most salient themes were related to (1) social isolation, (2) socio-emotional health of parents and child, and (3) financial strain (Table 3). In the theme of social isolation, some parents reported missing out on developmental experiences critical for their children’s growth and development and not being able to engage in social activities with family and friends. One parent said, “Family and friends are so important, it’s a crucial time for my daughter and the pandemic robbed that from her.”

For the theme of socio-emotional health, parents reported that the social isolation they experienced led to significant mental health changes in both themselves and in their children. They reported living with higher levels of stress, depression, anxiety, and fear. One parent shared, “It has been difficult for [the kids] and for all of us”, and another said “The pandemic made me very depressed… I felt that the world fell apart.” Another important theme that emerged was financial strain, with parents reporting loss of household income and resulting financial strain that could be attributed directly to the COVID-19 pandemic. One parent said, “I lost my job due to the pandemic… and I didn’t qualify for unemployment.” Although a majority of qualitative responses shared were in alignment with these salient themes, a few parents also reported positive impact of the pandemic on their household, including the ability to spend more time with family, eating home-cooked meals together, and improvements in parent–child relationships.

## 4. Discussion

Our findings indicate that parents with low-income households in the early childhood period during the pandemic suffered significant declines in mental and physical health and emotional support. The largest declines were for parental mental health, which is in alignment with parents’ qualitative descriptions of how the pandemic had impacted them and their family—specifically the challenges of dealing with social isolation and how that impacted their mental health. We also found that parent participation in a clinic-based community health worker intervention for early childhood well-child care visits buffered this observed negative impact on parental mental health but did not significantly buffer the decline in physical health or emotional support.

The vast majority of parents in our sample were low-income mothers in the first 24 months postpartum. The postpartum period, even without the pandemic, is a vulnerable period already associated with greater risk of worse maternal health [10,11]; the pandemic compounded these expected health and wellness declines for postpartum mothers [13,14,15,16]. However, less of a decline was seen for parent mental health in the intervention group, with a clinic-based community health worker that supports parents at early childhood preventive care visits, than the control group with usual well-child care. Other clinic-based, team-based preventive care models, such as *Healthy Steps for Young Children*, may similarly serve as important buffers for the health and wellness of postpartum mothers [17,18]. More robust funding models are needed so that clinics; particularly those that serve Medicaid-insured children can implement and sustain team-based preventive care.

It is unclear why the pandemic-era decline in parent mental health was buffered by the PARENT intervention, but emotional support and physical health were not. However, it may be that the PARENT coach was able to connect families to existing services for postpartum mental health needs, which tend to focus more on mental health promotion and treatment rather than the provision of emotional support or physical health programs. Additionally, for a majority of the parents who were Latina mothers, the PARENT intervention provided a bilingual, bicultural Latina community health worker available to them for support; this relationship may have also served as an important buffer that reduced the pandemic’s negative impact on parent mental health. While we do not have data to fully understand why certain parent health measures were buffered while others were not, we hypothesize that the resources, referrals, and support that the PARENT coach provided contributed to the differences.

A limitation of this study is our inability to differentiate the impact of the early childhood period to that of the pandemic; however, it is important to understand how these life events combined to create an impact on families. Non-response bias may be a concern; however, we obtained a response rate of 80% and observed only slight differences in country of birth and primary language spoken in the home. Another limitation is that we only included three items to assess parental health. Additional objective measures of health and well-being would strengthen the associations we found and could potentially help explain why parent mental health was buffered but emotional support and physical health were not. In addition, the study utilized one open-ended question for the qualitative analysis as opposed to a more extensive interview. The qualitative analysis is therefore limited to what families felt compelled to share with little to no prompting or exploration of the themes they shared.

In conclusion, our study documented, among a sample of primarily low-income mothers with young children, significant declines in mental and physical health and emotional support during the pandemic; we found that participation in a clinic team-based approach to preventive care visits using a community health worker buffered the declines in mental health. Our findings support the conceptualization that team-based care is essential to the provision of comprehensive, family-centered preventive care that meets the needs of families during early childhood [18]. Preventive care visits are a key touchpoint for families to receive support, resources, and services; team-based care can ensure that families receive that support and is an essential element in the provision of high-quality primary care [19].

## 5. Conclusions

Clinic-based interventions that are designed to support families with young children will be critical to buffer the ongoing health impact on families with low incomes, who are experiencing the added vulnerability of the early childhood period during the COVID-19 pandemic [19].

## Figures and Tables

**Table 1:** Baseline characteristics of survey respondents (Pre-COVID-19: March 2019–February 2020).

	Full Trial Population; n = 501 n (%)	Control Group; n= 266 n (%)	Intervention Group; n = 235 n (%)	*p*-Value *
**Child Age (months), mean (SD)**	4.6 (4.1)	5.1 (4.2)	4.1 (4.1)	0.012
**Participant relationship to child (mother)**	464 (92.8%)	245 (92.1%)	219 (93.6%)	0.52
**Participant Race and ethnicity**				0.17
Latino	333 (66.7%)	170 (64.4%)	163 (69.4%)	
Non-Latino Black	26 (5.2%)	13 (4.9%)	13 (5.5%)	
Non-Latino Asian	18 (3.6%)	10 (3.8%)	8 (3.4%)	
Non-Latino White	80 (16.0%)	45 (17.0%)	35 (14.9%)	
Non-Latino Multi-racial	12 (2.4%)	4 (1.5%)	8 (3.4%)	
Non-Latino Other	30 (6.0%)	22 (8.3%)	8 (3.4%)	
**Participant Language (primary)**				0.35
English	276 (55.1%)	154 (57.9%)	122 (51.9%)	
Spanish	184 (36.7%)	90 (33.8%)	94 (40.0%)	
Other	41 (8.2%)	22 (8.3%)	19 (8.1%)	
**Participant place of birth**				0.15
U.S.	280 (56.0%)	157 (59.0%)	123 (52.6%)	
Non-U.S.	220 (44.0%)	109 (41.0%)	111 (47.4%)	
**Child’s Health Insurance Type**				0.93
Medicaid (Medi-Cal/Apple Health)	482 (96.4%)	257 (96.6%)	225 (96.1%)	
Private Insurance	7 (1.4%)	4 (1.5%)	3 (1.3%)	
Uninsured	8 (1.6%)	3 (1.1%)	5 (2.1%)	
Unknown ^a^	1 (0.2%)	1 (0.4%)	0 (0.0%)	
More than one	2 (0.4%)	1 (0.4%)	1 (0.4%)	
Annual Household Income **				0.12
≤$29,999	227 (62.4%)	109 (56.8%)	118 (68.6%)	
$30,000 to $49,999	95 (26.1%)	56 (29.2%)	39 (22.7%)	
$50,000 to $69,999	24 (6.6%)	16 (8.3%)	8 (4.7%)	
≥$70,000	18 (4.9%)	11 (5.7%)	7 (4.1%)	
**PROMIS 2-item Global Mental Health T score, mean (SD)**	54.7 (8.4)	54.6 (8.2)	54.8 (8.8)	0.78
**PROMIS 2-item Global Physical Health T score, mean (SD)**	51.5 (8.0)	51.4 (7.7)	51.7 (8.4)	0.78
**PROMIS 4-item Emotional Support T score, mean (SD)**	58.0 (6.8)	57.7 (6.9)	58.2 (6.6)	0.42

^a^ Unknown insurance is for 1 participants who could not specify whether their insurance plan was through a commercial plan or Medicaid. * *p*-values used a two-sample *t*-test for continuous variables and Pearson’s chi-squared test for categorical variables. ** household income data were missing for 27.3% of the study population, including 27.8% of the control group and 26.8% of the intervention group (difference was not statistically significant; *p* = 0.80).

**Table 2:** Changes in Parent Global Mental Health, Physical Health, and Emotional Support from baseline (pre-pandemic: March 2019–February 2020) to 12-Month Follow-up (March 2020–February 2021), within study arms as well as differences across study arms.

	Mean (SD)						Adjusted Difference in Differences (95% CI) ^a,b^
	Control Group (n = 213)			Intervention Group (n = 188)			
	Baseline	12 Months	Difference from Baseline to 12 Months	Baseline	12 Months	Difference from Baseline to 12 Months	
**PROMIS Mental Health T-score**	54.1 (8.1)	50.8 (7.8)	−3.3 (8.7)	54.6 (8.6)	53.0 (8.0)	−1.6 (7.7)	**1.87 (0.26–3.49)**
PROMIS Physical Health T-score	51.2 (7.5)	49.7 (7.9)	−1.5 (8.3)	50.9 (8.4)	51.1 (7.7)	0.2 (8.7)	1.66 (−0.09–3.41)
PROMIS Emotional Support T-score	57.7 (6.9)	55.5 (7.8)	−2.2 (8.1)	58.3 (6.6)	56.2 (8.3)	−2.2 (8.1)	0.14 (−1.50–1.78)

^a^ Statistically significant differences are indicated by bolded values (*p* < 0.05). ^b^ Mixed effects linear regression with study arm as the independent variable and a random effect for clinic, adjusted for child age at baseline. PROMIS MH and PH were calculated by summing two five-point scale questions for a total possible of 2–10 (nine values), then converting those sums to corresponding PROMIS T-scores, for nine T-score values ranging from 25.8 to 64.6 (MH) and from 23.4 to 63.3 (PH). PROMIS Emotional Support was calculated by summing four five-point scale questions for a total possible of 4–20 (17 values), then converting those sums to corresponding PROMIS T-scores, for T-score values ranging from 25.7 to 62.0. https://staging.healthmeasures.net/images/PROMIS/manuals (accessed on 16 September 2021).

**Table 3:** Qualitative themes and illustrative quotes (August 2020–June 2021); “Is there anything else that you would like to share about how the COVID-19 pandemic has impacted you and your family?”

Theme	Illustrative Quotes
Social isolation	“The pandemic really sucks. Family and friends are so important, [it’s a] crucial time for my daughter and the pandemic robbed that from her. With masks she can’t see the expression on people’s faces, and her getting to know other children or family members, it got stolen from her.”
	“It’s been pretty isolating, especially as a family that just moved here.”
	“The distancing affected us because we are a very united family, and now we barely see each other. We have to now see each other over the phone.”
	“I can’t go out with my baby anymore, there has been a huge change with my routine. It’s a really sad situation. I would like for my baby to meet other babies, go outside, and learn. We have been isolated of many things.”
Socio-emotional health of parents and child	“The kids are depressed because they can’t go out. It has been really difficult for them and for all of us. They had to spend their birthdays inside.”
	“The pandemic made me very depressed. The month of March 2020 was a very difficult time where I felt that the world fell apart.”
	“It’s been hard for the kids. My 2-year-old doesn’t get to go anywhere at all, and it has caused her to be angry and defiant.”
	“For one of my kids it has been very difficult to concentrate in school. They feel very isolated, [and] they are not developing socially. My baby has also been affected in a way. He can’t be out exploring. I also can’t take him out to the library and my older children are not socializing.”
Financial Strain	“I lost my job due to the pandemic… and I didn’t qualify for unemployment.”
	“Money has been really hard. [I’m] afraid that I won’t be able to pay bills.”
	“There’s less work, and sometimes they don’t want us to go into work because of the pandemic.”
	“[I’ve] had issues purchasing food because of produce prices increasing due to the pandemic.”
	“Unemployment has been taking months and everything is backed up.”

## Data Availability

The data presented in this study are available on request from the corresponding author.
